# Supplementary material for: Essential Role of COPII Proteins in Maintaining the Contractile Ring Anchoring to the Plasma Membrane during Cytokinesis in Drosophila Male Meiosis
Source: Int J Mol Sci. 2024 Apr 20;25(8):4526. doi: 10.3390/ijms25084526 (PMC11050551; doi:10.3390/ijms25084526)
Supplement: Supplementary file 1 [file ijms-25-04526-s001.zip › ijms-2945278-supplementary.pdf]

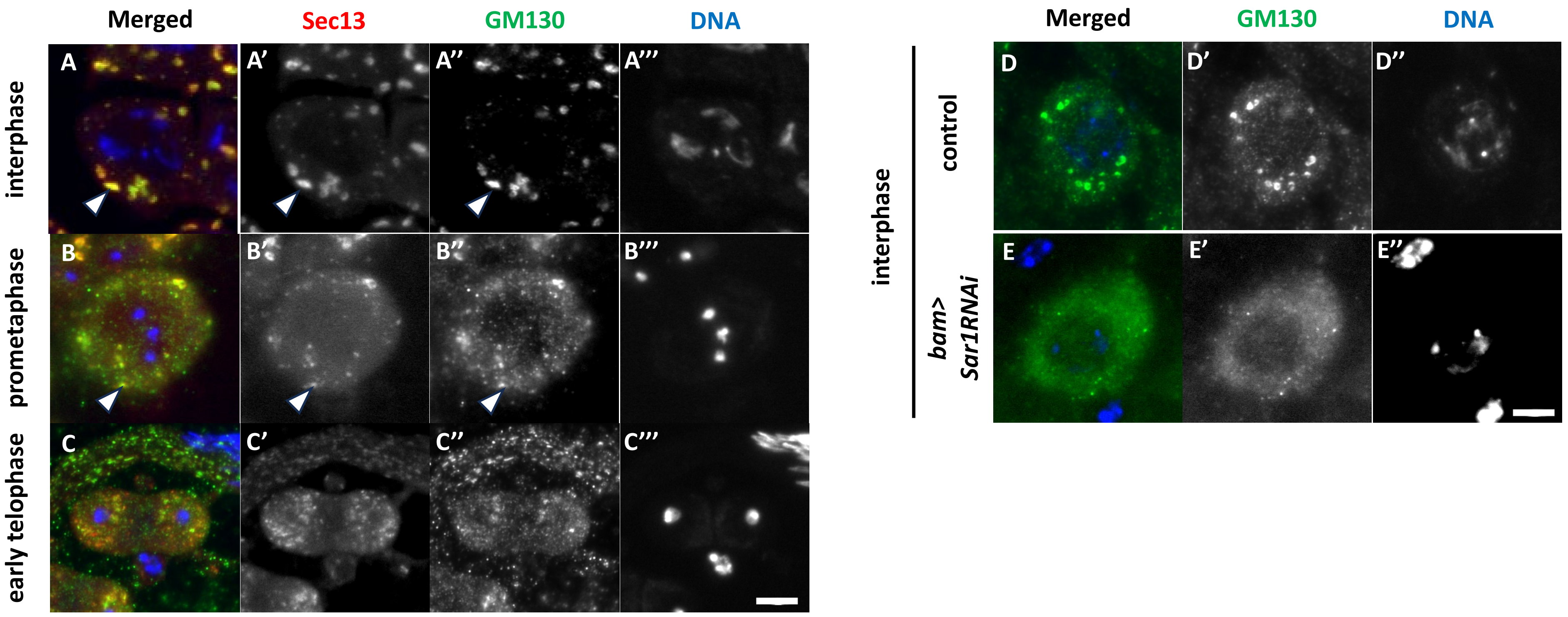
Merged
Sec13
GM130
DNA
interphase
prometaphase
early telophase
A
A'
A''
A'''
B
B'
B''
B'''
C
C'
C''
C'''
Merged
GM130
DNA
interphase
control
bam>
Sar1RNAi
D
D'
D''
E
E'
E''

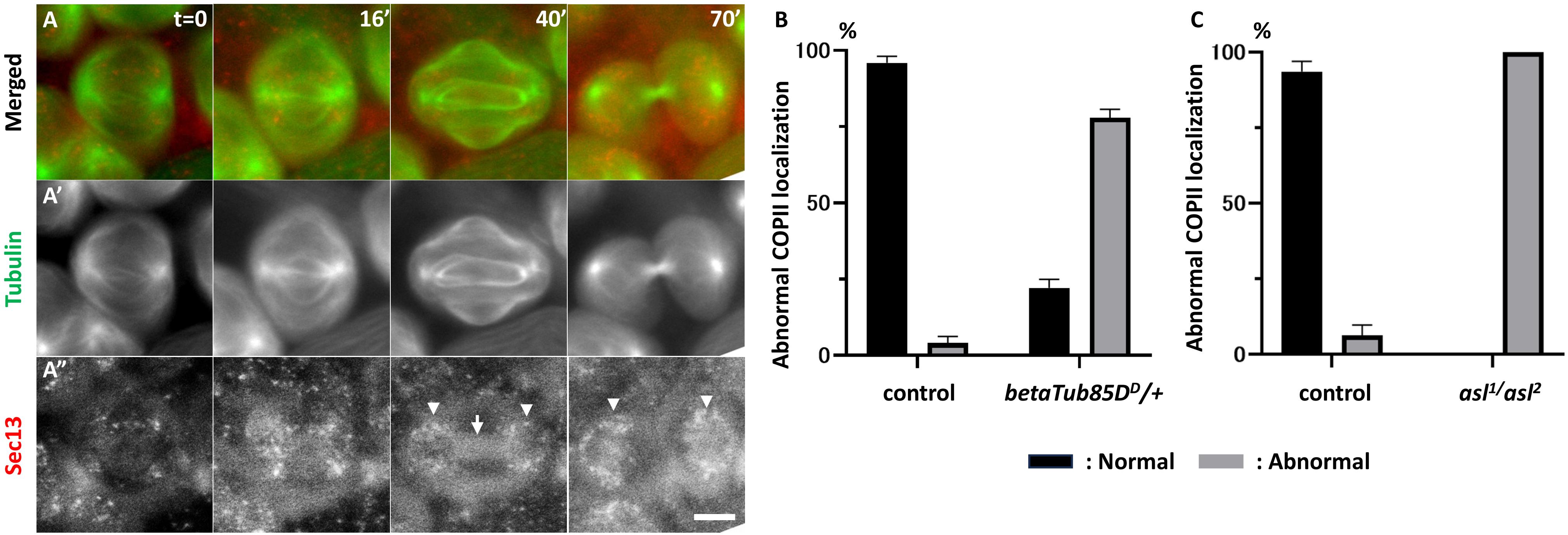
A
t=0
16′
40′
70′
A′
A″
Merged
Tubulin
Sec13
B
%
100
50
0
Abnormal COPII localization
control
betaTub85D^D/+
C
%
100
50
0
Abnormal COPII localization
control
asl^1/asl^2
: Normal
: Abnormal

Supplementary Figure Legends

Figure S1. Colocalization of the COPII foci with cis-Golgi marker before and during male meiosis I. (A-D) Anti-GM130 immunostaining (green in A to D, white in A" to H") of the primary spermatocytes expressing RFP-Sec13 before (S6 stage) (A) and those at prometaphase (B), early (C) and late (D) telophase. The RFP fluorescence is red in A to D, white in A" to H". Arrowheads in A and B indicate colocalization between Sec13 and GM130 foci. (E, F) Anti-GM130 immunostaining (green in E, F, white in E", F") of the control normal (*bam>+*) and *Sar1*-silenced (*bam>Sar1RNAi*) spermatocytes before (S6 stage) (A) and those at prometaphase (B), early (C) and late (D) telophase. DNA staining with DAPI (blue in A to D, white in A" to D"). Bars,10 µm.

Figure S2. Accumulation of COPII foci around astral microtubules until the end of meiosis and its disruption in spermatocytes homozygous for *testis-specific tubulin* or *asterless* mutation.
(A) Time-lapse observation of COPII foci (RFP-Sec13, red in A, white in A") and microtubules (GFP-Tubulin, green in A, white in A') during male meiosis I. The time when GFP fluorescence in spindle poles became distinctive is set as t=0' (prophase I). Based on the characteristics of the microtubule structure, meiosis progressed in this cell from the start point, metaphase I (t=16'), anaphase I (t=40') to cytokinesis (t=70'). Note that COPII foci become accumulated around the spindle poles (arrowheads) and inside of spindle envelop (arrow) in the cell (t=40') and are localized outside of astral microtubules at the end (t=70'). Bar,10 µm. (B, C) Quantification of meiotic cells showing the abnormal distribution of COPII foci in *betaTub85D* mutant testes (*GFP-Tubulin, Ub-RFP-Sec13/+;betaTub85D^{D}/+*) and normal control testes (*GFP-Tubulin, Ub-RFP-Sec13/+* (B), and in *asl* mutant testes (*GFP-Tubulin, Ub-RFP-Sec13/+; asl^{1}/asl^{2}*) and normal control testes (*GFP-Tubulin, Ub-RFP-Sec13/+*). The bars represent the SEM.
